# Material and Structural Modeling Aspects of Brain Tissue Deformation under Dynamic Loads

**DOI:** 10.3390/ma12020271

**Published:** 2019-01-15

**Authors:** Monika Ratajczak, Mariusz Ptak, Leszek Chybowski, Katarzyna Gawdzińska, Romuald Będziński

**Affiliations:** 1 Faculty of Mechanical Engineering, University of Zielona Góra, 65-516 Zielona Góra, Poland; r.bedzinski@ibem.uz.zgora.pl; 2 Faculty of Mechanical Engineering, Wrocław University of Science and Technology, 50-370 Wrocław, Poland; 3 Faculty of Marine Engineering, Maritime University of Szczecin, 70-500 Szczecin, Poland; k.gawdzinska@am.szczecin.pl

**Keywords:** biomechanics of the brain, brain injury process, mechanical properties of brain tissue, numerical simulation, finite element method (FEM), fluid-elastic materials, viscoelastic materials, hyperelastic materials, dynamic response

## Abstract

The aim of this work was to assess the numerous approaches to structural and material modeling of brain tissue under dynamic loading conditions. The current technological improvements in material modeling have led to various approaches described in the literature. However, the methods used for the determination of the brain’s characteristics have not always been stated or clearly defined and material data are even more scattered. Thus, the research described in this paper explicitly underlines directions for the development of numerical brain models. An important element of this research is the development of a numerical model of the brain based on medical imaging methods. This approach allowed the authors to assess the changes in the mechanical and geometrical parameters of brain tissue caused by the impact of mechanical loads. The developed model was verified through comparison with experimental studies on post-mortem human subjects described in the literature, as well as through numerical tests. Based on the current research, the authors identified important aspects of the modeling of brain tissue that influence the assessment of the actual biomechanical response of the brain for dynamic analyses.

## 1. Introduction

Traumatic brain injury (TBI) is a highly severe condition, of which the main cause is excessive mechanical loading occurring during vehicle accidents, sports injuries, violence, or injuries related to everyday activities (e.g., impact with furniture or falling down stairs). The effect of these injuries can include cerebrovascular damage, neuronal deformation, hypoxia, cerebral edema, and increased intracranial pressure. Destructive changes to brain tissue caused by external mechanical loads are the cause of serious neurological and neurobehavioral disorders [1,2]. 

Craniocerebral injuries can result in permanent disability and complete exclusion from social activities, which is associated with the need for long-term treatment and high costs for a country’s health system. According to the American Association of Neurosurgeons, the direct and indirect costs of TBI treatment in the United States of America amount to 48–56 million dollars annually [3]. The effects of TBI can occur immediately after the injury in the form of local pain and disturbances in neuromotor function, or may appear long after the original injury. The latter changes may manifest as progressive dementia disorders, such as Alzheimer’s disease. 

The effects of TBI are not only difficult to diagnose in their early stages but are also often underestimated by medical staff. Currently, in addition to neurophysiological testing, the best way to explore disorders of the central nervous system (CNS) is by using diagnostic imaging methods, such as magnetic resonance imaging (MRI) and computer tomography (CT) [4]. However, these methods do not provide an insight into the biomechanical behavior of the brain tissues. Thus, damage to brain tissues needs to be further researched. At the same time, the most up-to-date literature has suggested that the main mechanisms of disorders of brain structure and function, including neurological diseases, are directly related to the mechanical strain thresholds of the brain’s structures being exceeded. 

To date, the mechanisms of brain tissue degradation have neither been precisely identified nor described. One of the best methods to identify the responses of biological structures to loads is through numerical modeling [5,6,7,8,9,10]. Therefore, with the exponential growth of computational power, there has been an increasing interest in the use of the finite element method (FEM) for structural modeling of the human head [11,12,13,14,15,16,17,18,19,20]. The constitutive material models of brain tissue and the finite element analysis of blast-induced traumatic brain injury have been discussed in the literature [21,22,23,24,25,26]. So far, many numerical models of the head have been developed and this topic is still being expanded upon. 

However, only a few of these models have been verified by comparison with experimental studies carried out on post-mortem human subjects (PMHS). In addition, most papers in the literature carried out analyses of the mechanical response of brain tissues using shared finite-element nodes between the brain and skull models; nevertheless, modeling the skull-brain connection in this way does not allow a complete understanding of the biomechanics of the head. At the same time, there is no clear consensus in the literature on how to model brain tissue [27,28]. 

On the other hand, the choice of the material of the model can affect the brain kinematics in relation to the skull. Moreover, only a few numerical models have included bridging veins [16,29,30,31,32,33,34]. The authors of these models stated that the inclusion of these elements may significantly affect the response of the entire brain-tissue system. Taking into account the fact that trauma criteria aim to reduce the incidence of TBI, it is necessary to develop a reliable and computationally-efficient numerical model of the human CNS system. As a result of a literature review, the main purpose of this work was to develop a numerical model of the head that mimics the actual behavior of brain tissue during mechanical loading. For this purpose, the authors evaluated the influence of the modeling method of the brain tissue system on its biomechanical response with reference to experimental studies, which were carried out on PMHS [35].

## 2. Methods

### 2.1. Model Generation

A 3D geometric model of the brain and skull was created based on medical images acquired from medical scanners. The 3D object that was created was exported in the stereolithography format (STL), which enabled the authors to proceed with digital processing using computer programs such as CATIA v5 (Dassault Systèmes, Vélizy-Villacoublay, France) and MeshLab (Institute of Information Science and Technologies—Italian National Research Council, Pisa, Italy). The geometry of the brain was divided into four parts: the right and left hemispheres of the brain and the right and left parts of the cerebellum. 

**Figure 1:**
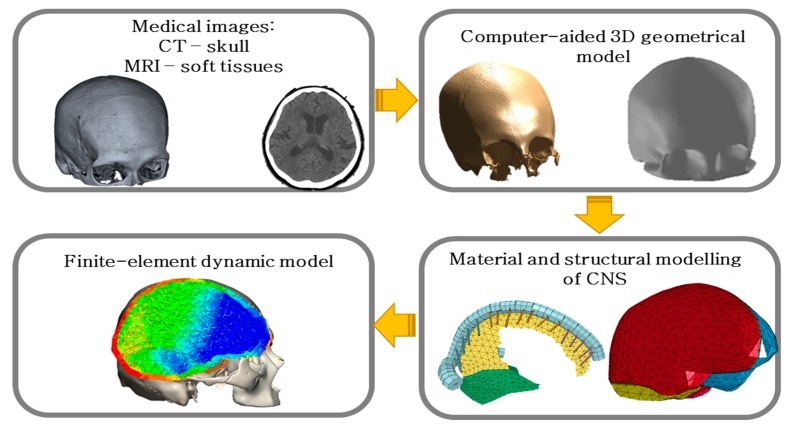
Procedure for creating a numerical model of the head.

The model also included advantageous features in terms of biofidelity, such as dura mater, cerebrospinal fluid (CSF), falx cerebri and cerebellar tentorium, superior sagittal sinus, and bridging veins. The design of the bridging veins was based on the literature [16,36,37]. In the numerical model, the bridging veins were distinguished between the frontal, parietal, and occipital parts. This model was named αHEAD (Figure 1). 

The conversion of the geometrical model to the finite-element discrete system was carried out in ANSYS (Ansys, Inc., Canonsburg, PA, USA). Particular attention was paid during the meshing process to the quality of the finite element mesh using the mesh-checking tool in ANSYS. Further, the authors transformed the finite element (FE) model to LS-DYNA (Livermore Software Technology Corporation, Livermore, CA, USA) explicit code. Due to the irregular shape of the brain, skull, and cerebrospinal fluid, these structures were meshed using tetrahedral elements. Thus, the model consisted of solid finite elements (cerebrum, cerebellum, skull, and CSF), shell elements (dura mater, falx cerebri, cerebellar tentorium) and beam elements (superior sagittal sinus, bridging veins, and outflow cuff segment). 

The CSF was modeled using the fluid-elastic material type. The remaining elements of the brain were modeled as elastic elements (except for the central nervous system). In the numerical model, connections were established between the structures, which enabled the model to mimic the brain’s natural movement under mechanical loads. Based on the work of Horgan and Gilchrist [38] and Miller et al. [39], the static and dynamic friction coefficients were set to 0.15 and 0.20, respectively, for the structures that were in contact with each other. The mechanical properties measured in standard conditions and used in this study are summarized in Table 1 and Table 2.

### 2.2. Constitutive Modeling of Central Nervous System

There is no unambiguous and accepted consensus in the literature about the modeling methodology of the CNS [42,43]. Brain models with linear-elastic, viscoelastic, or hyperelastic materials can be found in these studies. Among the hyperelastic material models, the Ogden and Mooney-Rivlin models have been used most often. In this study, two different viscoelastic models, three Mooney-Rivlin models with different stiffness parameters, and Ogden’s model were used. The expression describing shearing for the viscoelastic material for the brain can be represented by the equation:

(1) $$G(t)=G_{\infty }+(G_{0}-G_{\infty })e^{-\beta t}$$

where *G*_∞_ is the shear modulus after an infinitely long time, *G*_0_ is the shear modulus for *t* = 0, *β* is the decay coefficient, and *t* is time.

The values of the individual coefficients used in the study for the viscoelastic model are summarized in Table 3.

The simplified expression of the Mooney-Rivlin model for computations using the finite element method can be given by the equation:

(2) $$g(t)=\sum_{i=1}^{n}\left(G_{i}e^{-\beta_{i}t}\right)$$

where *G_i_* is the shear modulus of the *i*-th element. As *G_i_* = 2(C_10_ + C_01_), the C_10_, C_01_ constant values given in Table 4 were used in the study.

The shear modulus for the Ogden model for numerical calculations can be represented in the following form:

(3) $$G=\frac{1}{2\sum_{i=1}^{n}\left(\alpha_{i}\mu_{i}\right)}$$

where *α_i_* and *μ_i_* are Ogden’s parameters with values as follows: *α*_1_ = 10.1, *α*_2_ = −12.9, *μ*_1_ = 53.8 Pa, *μ*_2_ = −120.4 Pa.

The constitutive model and corresponding parameters were adjusted based on the work of Franceschini et al. [49], while the high frequency relaxation modules were determined on the basis of Nicolle et al. [50].

### 2.3. Volumetric Locking in Numerical Model

In explicit dynamic FEA applications, when simulating almost incompressible structures, and where Poisson’s ratio is close to 0.5, the stiffness of the fully integrated finite elements is often overestimated. As a consequence of this, the results may become unrealistic and the model will thereby become invalid, as the locking of these elements produces severe spurious fluctuations in the hydrostatic pressure. Inauthentic pressure stresses develop at the level of the element’s integration point, causing the finite element to behave in too stiff a manner for deformations that should induce no volume change. 

This effect has been described in the literature under the term *volumetric locking*. Van den Oord’s research proved that this phenomenon occurs most often with tetrahedral four-node elements [51]. The brain tissues as well as the CSF are structures with a Poisson’s coefficient that is close to 0.5. Consequently, the locking phenomenon often appears in numerical analyses [52,53]. The use of the ELFORM13 (i.e., element formulation options (section), available in LS-DYNA) enabled the elimination of the volume locking phenomenon in this paper. Volumetric locking is usually seen in the post-processor while displaying the hydrostatic pressure stress at the integration points as a checkerboard mode. The ELFORM13 element formulation options prevented volumetric locking in the model by defining the nodal volumes and evaluating the average nodal pressures in terms of these volumes. Nevertheless, it must be restated that the tetrahedral element is significantly less computationally efficient than the standard hexahedral elements. Thus, it should only be used when the initial geometry cannot be meshed with hexahedra. The authors are aware that with the current technology level, the complex geometry of the human head may preclude researchers from achieving semi-automatic hexahedral mesh generation, as this is a challenging issue.

### 2.4. Boundary Conditions and Material Verification 

**Figure 2:**
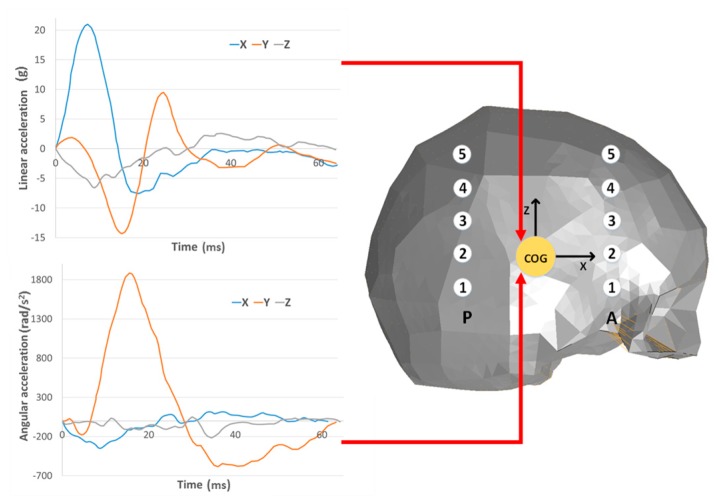
Boundary conditions for Hardy et al. [35] with marker numbers for the frontal (A) and rear columns (P).

The boundary conditions for the simulations were set in accordance with Hardy et al. [35], where the measurements were recorded using a 6-axis accelerometer, which registered both linear and angular acceleration. The output data from the accelerometer, with six different acceleration functions in time, was used in the numerical simulations as the input load function applied to the skull’s center of mass (Figure 2).

The geometric and material verifications were carried out based on the correlation with Hardy’s research [35], which was based on the measurement of the relative displacement of the brain in relation to the skull. The test with the identifier C755-T2 was selected for this research. This test was chosen because of the similar dimensions of the head to the developed numerical model. In this study, the effect of the selection of the material model on the relative displacement of the brain was investigated. In the last step, the validated results of the αHEAD model were compared to the results of YEAHM (YEt Another Head Model), which were validated using the same test C755-T2 [12].

## 3. Results

**Figure 3:**
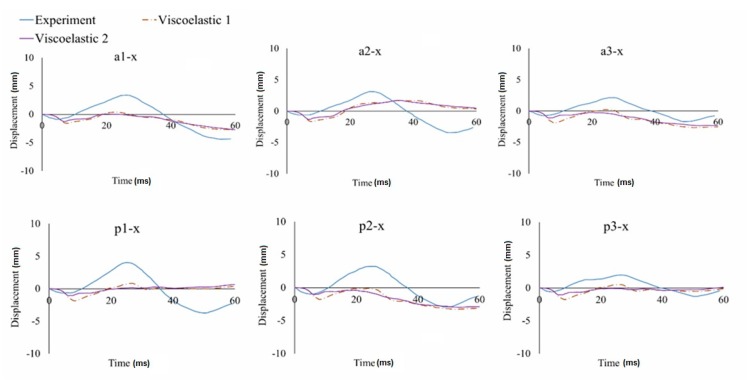
Displacement-in-time characteristics of the markers in the frontal (a) and rear columns (p) in the X-direction using the viscoelastic material models approach.

**Figure 4:**
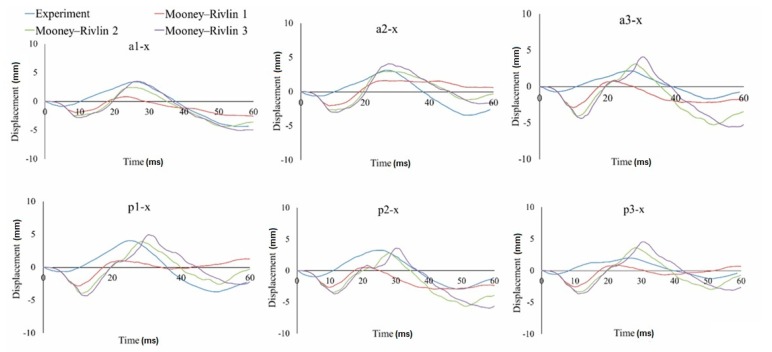
Displacement-in-time characteristics of the markers in the frontal (a) and rear columns (p) in the X-direction using the hyperelastic material models based on the Mooney-Rivlin constitutive equations.

**Figure 5:**
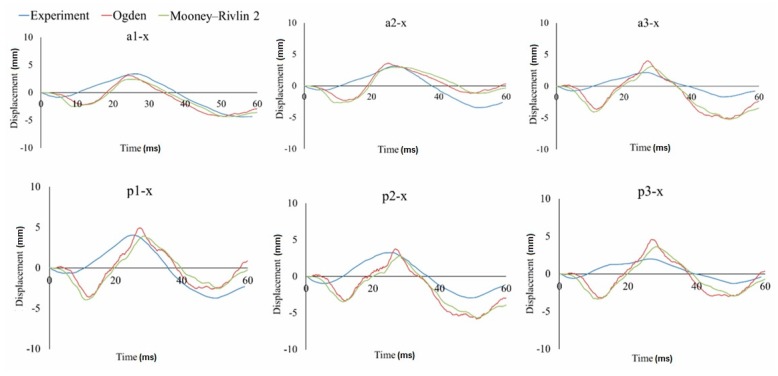
Displacement-in-time characteristics of the markers in the frontal (a) and rear columns (p) in the X-direction using the hyperelastic material models based on the second Mooney-Rivlin and Ogden constitutive equations.

In this study, the relative displacement of the brain was analyzed in relation to the skull with five markers in the X (sagittal axis) and Z (longitudinal axis) directions for the occipito-parietal and tempero-parietal regions [54]. The markers were used to evaluate the properties of the different material models to reproduce the relative brain displacements obtained from Hardy’s C755-T2 experiment [35]. Figure 3, Figure 4 and Figure 5 illustrate the characteristics for selected markers in the frontal column (A) and rear column (P), with each graph representing the selected marker locations. On all of the graphs, characteristic minima occurring between 0–20 ms and maxima between 20–40 ms can be observed. The results for the two chosen viscoelastic material models are depicted in Figure 3. The results for the three different Mooney-Rivlin models are shown in Figure 4. The results of the comparison of the second Mooney-Rivlin material model with the Ogden model are shown in Figure 5. Another alternative for reporting the validation results would be the CORA (CORrelation and Analysis) method [55,56]. All the material models in the following graphs were contrasted with the output of the C755-T2 experiment [35].

**Figure 6:**
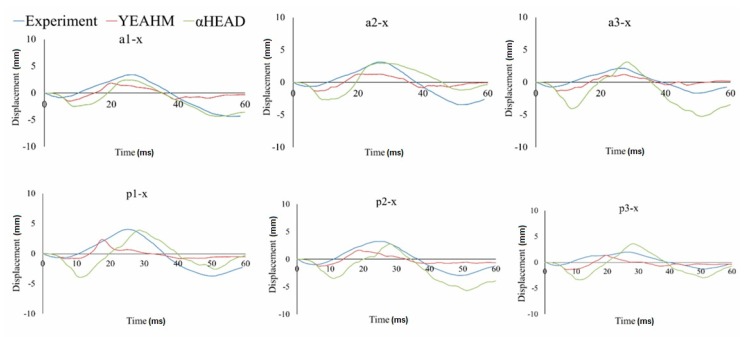
Comparison of the displacement-in-time characteristics of the markers in the frontal (a) and rear columns (p) in the X-direction for the C755-T2 test, and αHEAD and YEAHM numerical models.

Further, the authors compared the validated results of αHEAD with YEAHM (YEt Another Head Model). The results from the YEAHM model have been previously compared with the results of Hardy et al. [35]. The author of the YEAHM, summarizing the results of its validation, stated that the worst fit of the characteristics was reported for the markers a1, a2, p1, and p2 in the X-direction. This conclusion was based on the lack of a falx cerebri and cerebellar tentorium in the YEAHM model [57]. Based on the results obtained from the αHEAD model, which included the falx cerebri and cerebellar tentorium, it could be concluded that this assumption was reasonable. It can be seen from Figure 6 that in the αHEAD model, the best fit of the characteristics to the experiment was obtained for the markers a1, a2, p1, and p2 in the X-axis. 

These markers were located a short distance from the cerebellar tentorium and the lower part of the falx cerebri; therefore, these structures had a significant impact on the brain’s overall displacement. However, in the αHEAD model, the worst match was found for the markers located in the upper layers of the brain (e.g., a5-x and p5-x). This was associated with the lack of gyri and sulci in the brain model. This conclusion could be confirmed by comparing the results of the YEAHM (which included the grooves and ridges in the cerebral cortex) with the Hardy et al. [35] experiment, where a good correlation for the a5-x and p5-x markers was obtained. The confirmation of this phenomenon can be found in the studies of Ho and Kleiven [58]. They concluded that the inclusion of these structures had a significant impact on reducing the deformation distortion of the brain tissues during a TBI. In vertebrates, the characteristic folds of the cerebral cortex, created during the gyrification process, arose due to the evolutionary growth of the neurons in the neocortex. Interestingly, as was figured out in these studies, the gyri and sulci may have a significant impact on the brain’s response to mechanical loads.

## 4. Discussion

Taking into account the displacement runs of all the markers in the front and rear columns, the best correlation with the experiment was obtained for the second Mooney-Rivlin material model with constants C_10_ = 62 and C_01_ = 69, as well as for the Ogden material model. The best curve match was evaluated in MATLAB using the least squares regression analysis. Based on the studies of the three stiffness parameters for the Mooney-Rivlin model, it could be noticed that the size of the brain’s relative movement increased with decreasing stiffness of the brain tissue. Similar conclusions were drawn by Kleiven [59].

While using viscoelastic models, relatively small brain displacements in the X-direction were obtained. Therefore, for the a3-x, a5-x, p3-x, p4-x, and p5-x markers, a good convergence with the experiment was acquired. However, for the markers that were located close the cerebellar tentorium, the results were less consistent with the experiment. Takhounts [18] also validated the SIMon model on the C755-A2 test using a viscoelastic material model—similar to this study—and registered very small displacements for the markers located close to the cerebellum tent. 

Comparing the hyperelastic and viscoelastic material models, it was found that both models showed a good correlation with the experiment. Nevertheless, in the case of the viscoelastic models, the relative displacement of the brain was lower than in the hyperelastic models. In addition, the relative displacement of the brain (markers) for the X component varied significantly over time in the hyperelastic models. As a result, a characteristically rapid change of the displacement between 5 and 20 ms (for the X-axis) could be observed. This distinctive displacement was related to the brain’s inertia and the shift of the brain’s mass towards the occipital bone. This effect was specific to the coup and contre coup phenomenon. Therefore, the model was validated using the second Mooney-Rivlin material model. 

**Figure 7:**
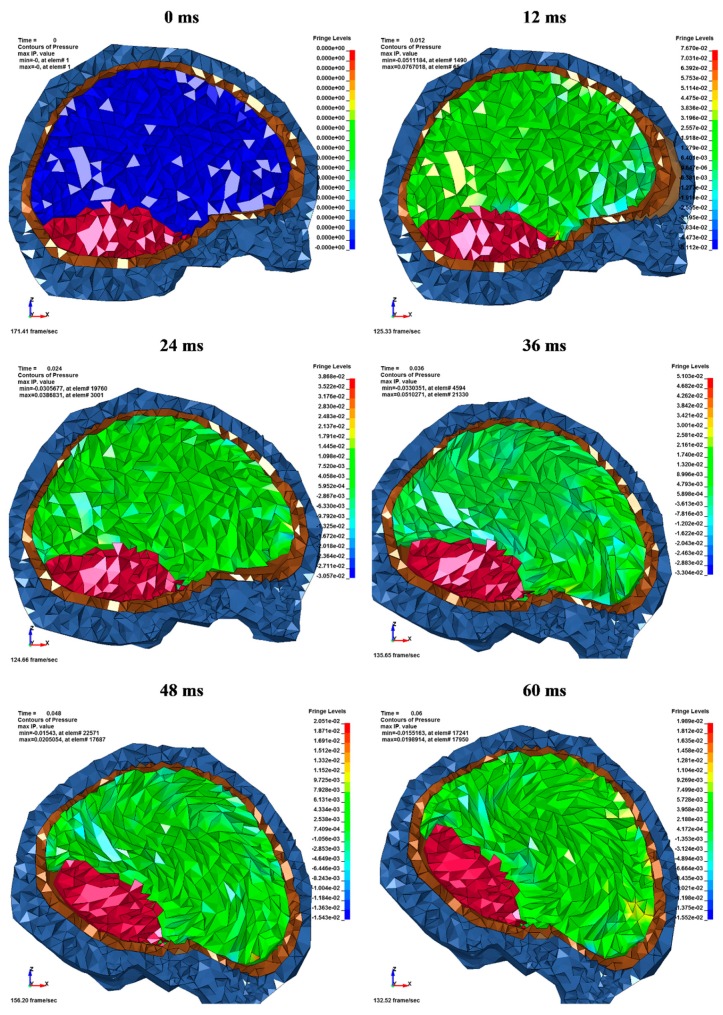
Head kinematics during the C755-T2 numerical test, with the cross-section in the median plane and showing the hydrostatic pressure (MPa) in the cerebrum.

Despite there being some differences in the results obtained by Hardy et al. [35] and those computed in these simulations, the course of the acquired characteristics in the model were close to those of the experiment. Figure 7 depicts the kinematics of the head during the numerical simulation of the C755-T2 test with a map of the hydrostatic pressure in the cerebrum. A discussion regarding the relationship between axonal strain and brain tissue deformation has not been provided here as it is not directly connected to the main aims of this experiment. Additional information on this issue has been presented in the literature [60,61].

It should be noted that, unlike the YEAHM, the αHEAD numerical model does not have cerebral ventricles, in which the CSF is produced. Ivarsson et al. [62] found that the CSF reduced the deformation in the regions around the ventricular system. This was also confirmed by Kleiven [63], who observed a low level of deformation of the brain tissues in the proximity of the ventricles. Moreover, the differences between the experiment and the outcomes of the simulation may result from individual anthropometric features of the head, such as the geometry of the skull. Arcot and Genin [64] suggested that the shape of the skull has an important impact on the damage to brain tissue during mechanical injuries. Therefore, it could be concluded that cranial geometry may play an important role in the displacement of the brain tissues under impact load.

The numerical model did not contain structures such as pia mater, foramen magnum, or an irregular base of the skull. Ivarsson et al. [62] found that anatomical irregularities on the base of the skull protected the nerves and vessels running through the brain by reducing the displacement of the brain itself. Furthermore, other limiting factors affecting the results may be the lack of roughness in the inner part of the skull, the lack of mandible in the model, and not including geometrical changes in the structure of the brain due to the age of the PMHS. It should be emphasized that the description of the experiment by Hardy et al. [35] lacks information on whether the examined person had tauopathy (e.g., Alzheimer’s disease) or any similar neurological pathologies. These disorders are common among the elderly [65,66], significantly affect the physical structure of brain tissue, and can be crucial in the assessment of the brain’s relative movements. However, the current numerical head models presented in the literature, which have been validated with Hardy et al. [35], have usually included the geometry of a healthy brain without any neurodegenerative changes. 

Living brain tissues are highly vascularized, and the role of blood flow and pressure on the tissue’s behavior remains to be studied in future work. Nevertheless, the numerical αHEAD model of the human head developed in this work may improve the biomechanical criteria as well as head-protective systems based on new materials [67,68], facilitating the reconstruction of accidents in terms of forensics, and support the acquisition of knowledge in the field of head injuries. This research makes an important contribution to the biomechanical analysis of traumatic brain injuries and the development of numerical models of the human head.

## 5. Final Conclusion

The core findings of this paper are as follows. An examination of brain displacement and deformation during impact was performed, the results of which were validated based on data from Hardy et al. [35]. Compared to other numerical head models validated with tests by Hardy el al. [35], the authors achieved very good compatibility. Moreover, the brain’s material properties were modeled using the hyperelastic and viscoelastic constitutive law. Based on this analysis, it was found that the Mooney-Rivlin model was the most suitable for studying the biomechanical response of the brain.

## Figures and Tables

**Table 1:** Mechanical properties for each component of the head [21,40].

Element	Young’s (E) or Bulk Modulus (K) (MPa)	Density ρ (kg/m^3^)	Poisson’s Ratio ν
Skull	E = 15000.0	2000	0.22
Dura mater	E= 31.5	1130	0.45
Cerebrospinal fluid	K = 2200.0	1000	0.49
Superior sagittal sinus	E= 28.2	1040	0.45
Falx cerebri	E = 31.5	1130	0.45
Cerebellar tentorium	E = 31.5	1130	0.45

**Table 2:** Mechanical properties of bridging veins (samples from individuals older than 50 years of age) [41].

Bridging Veins Region	Young’s Modulus E (MPa)	Density ρ (kg/m^3^)	Poisson’s Ratio ν
Frontal	56.45	1130	0.45
Parietal	94.09	1130	0.45
Occipital	97.21	1130	0.45

**Table 3:** Values of individual coefficients used in the current study.

Model Name	Reference	Bulk Modulus K (MPa)	*G*_0_ (MPa)	*G*_∞_ (MPa)	*β* (s^−1^)
Viscoelastic 1	Shuck and Advani [44]	2190.00	0.528	0.1680	35
Viscoelastic 2	Willinger et al. [45]	1130.00	0.049	0.0167	145

**Table 4:** Parameters of the Mooney-Rivlin model used in the current study.

Model Name	Reference	C_10_ (Pa)	C_01_ (Pa)
Mooney-Rivlin 1	Mendis et al. [46]	620.5	689.4
Mooney-Rivlin 2	Arbogast and Margulies [47]	62.0	69.0
Mooney-Rivlin 3	Prange et al. [48]	31.0	35.0
